# Polarisation changes in guided infrared thermography using silver halide poly-crystalline mid-infrared fibre bundle

**DOI:** 10.1007/s10973-020-10018-0

**Published:** 2020-07-22

**Authors:** Sarah K. Markham, Aladin Mani, Elena A. Korsakova, Aleksandr S. Korsakov, Liya V. Zhukova, Joanna Bauer, Christophe Silien, Syed A. M. Tofail

**Affiliations:** 1 Department of Physics and Bernal Institute, University of Limerick, Limerick, Ireland; 2 Ural Federal University named after the first President of Russia B. N. Yeltsin, Ekaterinburg, Russia; 3 Department of Biomedical Engineering, Faculty of Fundamental Problems of Technology, Wroclaw University of Science and Technology, Wrocław, Poland

**Keywords:** Infrared thermography, Optical polarisation, Silver halide poly-crystalline, Broadband mid-infrared fibre bundle, Personalised medicine

## Abstract

Broadband mid-infrared (B-MIR) thermography using fibre optic waveguides can be critical in real-time imaging in harsh environments such as additive manufacturing, personalised medical diagnosis and therapy. We investigate the polarisation effect on thermal measurements through poly-crystalline fibre bundle employing a simple broadband cross-polarisation configuration experimental set-up. Silver halide poly-crystalline fibres AgCl_1−x_Br_x_ (0 ≤ *x*≤1) (AgClBr-PolyC) have very wide transmission bandwidth spanning over the spectral range from 1 µm up to 31 µm FWHM. Moreover, they are non-toxic, non-hygroscopic, with relatively good flexibility, which make them very adequate for spectroscopic and thermal measurements in medical and clinical fields. In this study, we used a fibre bundle composed of seven single AgClBr-PolyC fibres, each with a core diameter of about 300 µm, inserted between two broadband MIR polarisers. A silicon carbide filament source was placed at the entrance of the fibre bundle, while a FLIR thermal camera with a close-up lens was employed to measure the spatial temperature distribution over the fibre-bundle end. Indeed, polarisation dependence of temperature measurements has been clearly observed in which the orientation of temperature extrema (minima and maxima) vary from one fibre to another within the bundle. Moreover, these observations have enabled the classification of AgClBr-PolyC fibres following their polarisation sensitivities by which some fibres are relatively highly sensitive to polarisation with polarisation temperature difference (PTD) that can reach 22.1 ± 2.8 °C, whereas some others show very low PTD values down to 3.1 ± 2.8 °C. Many applications can readily be found based on the advantages of both extreme cases.

## Introduction

Infrared thermography (IRT) has been widely recognised in the medical field as an accurate and a relatively simple in vivo imaging technique. For example, the Food and Drug Administration (FDA) of the USA has approved IRT in 1982 as an adjunctive tool in breast cancer diagnosis [1] to provide additional functional information on the thermal and vascular condition of the tissues [2]. Imaging of external body parts [3, 4] has so far dominated medical applications of IRT including breast and other cancer detection [5], dermatology [6–8], burn trauma [9], diabetic neuropathy and peripheral vascular disorders [10].

By contrast, IRT for internal body in vivo medicine has proven relatively difficult due to a lack of transmission system thin enough to be manoeuvred through endo-vascular access and capable of imaging thermal variations in internal tissues with sufficient pixel size and spatial resolution. Mid-infrared (MIR) power sources, fibre delivery systems, sensors and detectors have been limiting factors in the adoption of medical mid-infrared imaging [6, 11].

Amongst these constraints, suitable MIR waveguides are very important for transmitting thermal emission, e.g., from an internal tumour site of high metabolic activity and thermal energy to an external imaging system. MIR transmission is required as near body temperature thermal emissions usually peak in the vicinity of 9–10 µm. This wavelength falls within the MIR region [12], typically defined to range between 2.5 and 20 µm. Fibres capable of transmitting such a wide range are known as broadband MIR (B-MIR) fibres. Saito and Kikuchi [13] list efforts made in using MIR waveguides in remote temperature measurements including thermal imaging for biological and medical applications such as urinary stones diagnosis, tissue welding and tissue and skin radiometric measurement. Later on, also more advanced trials were reported, e.g., real time in situ thermometry inside an MRI system [14], liver disease severity assessment including the metabolic alterations like liver failure, cirrhosis or portal hypertension [15], metabolic investigation of tumourous tissues [16, 17] or human cells [18, 19]. IRT has already been shown to be advantageous in the determination of the location, size, shape and margin of cancerous lesions [20] and its early diagnosis [11, 20]. Since IRT camera allows fast and non-contact measurements of spatial variation in temperature, and the emissivity of live tissue is near unity due to the high content of water in biological system, coupling bundle B-MIR fibre with an IRT camera could be hugely beneficial in endoscopic examination of internal tissues. Thermal imaging can be further utilised to record thermal behaviour of tissues in response to irradiation by, for example, an MIR laser, the power of which can also be transmitted through the same waveguide.

There has been an increase in the development of components in the last number of years to fill the gap in mid-infrared thermography, such as the advancement of silver halide fibres [21–23]. Infrared fibres come in many forms such as solid core fibres, hollow fibres and photonic crystal fibres, and a range of materials can be used [24, 25]. For medical applications, silver halide-based fibres stand out amongst the rest. They are an ideal candidate for the transmission of mid-infrared wavelengths from 2 to 20 μm [26–29]. Additional benefits of silver halide fibres for personalised, internal medical applications include mechanical flexibility, non-hygroscopicity and a lack of toxicity [30].

The application of polarised light in the visible is quite common in histopathology and diagnostic imaging [31–33]. Recently, this has been extended to infrared imaging, where the use of polarised infrared light had made cancerous features visible that had been undetectable when an unpolarised infrared light had been used [30]. Temperature-dependent birefringence of specific facets of single crystal lithium tantalate crystals (LiTaO_3_) has been used in thermometry [34]. However, the occurrence of polarisation change during waveguiding is relatively rare. In this article, we report changes in polarisation during infrared thermography using MIR poly-crystalline, silver halide-based bundle fibres. The changes in MIR polarisation occurring during waveguiding through the bundle fibres were detected as temperature variations in an infrared camera.

Silver halides, as a bundle fibre waveguide, have not received attention. Photonic crystal fibres (PCF) [35] and strip waveguides [36] made of silver halide materials have been reported for MIR waveguiding represent entirely different technologies to bundle fibre waveguide technology in terms of geometrical structure and waveguiding. In PCF, MIR is guided through an effective core bound by the photonic structure of inserts. Thus, there is a single waveguide channel. In the strip waveguide, MIR is guided along the strip which is placed on a AgCl/Ag/Ti/SiO_2_/Si wafer. There is only a single waveguide channel. In contrast, in our bundle fibre waveguide, MIR is guided through every fibre of the bundle, which means that there are now as many light guide channels as are the number of the fibres in the bundle. None of these silver halide waveguides reported changes in polarisation changes in the MIR wave that propagates through these waveguides.

In our study, we thus make a step towards the realisation of polarimetric thermal imaging through silver halide bundle fibres which can be advantageous in many types of thermal measurements of inaccessible areas using infrared waveguiding [37]. For example, conventional passive infrared imaging systems rely on detecting intensity contrast, which is often limited by low signal-to-noise contrast, giving rise to a small temperature contrast between normal and precancerous tissues in thermographic detection of early-stage cancer. To improve signal-to-noise contrast and, thereby, to reduce the probability of false detection, polarisation effects can act as an additional discriminator and make multimodal use of silver halide bundle fibres [38].

The novelty and significance of our work can be highlighted as follows:

1. We used a simple set-up to study and demonstrate polarisation effect on remote thermal measurements using broadband infrared bundle fibre.
2. We showed and classified for the first time two distinct types of broadband bundle fibres:
  - polarisation-sensitive fibres
  - polarisation-insensitive fibres
  Polarisation-sensitive fibres can be good candidates for polarisation-maintaining fibre applications. Polarisation-insensitive fibres can be good candidates for polarisation-free fibre applications. No such distinctions have so far been made in mid-infrared fibres.
3. Polarisation effects in visible light microscopy are an established technique in clinical practices such as histopathology and cytology. Our new finding of polarisation effect in thermal transmission through broadband bundle fibre can open up new opportunities in thermographic diagnoses towards more personalised and preventive diagnosis as well as therapy.

## Experimental

**Fig. 1:**
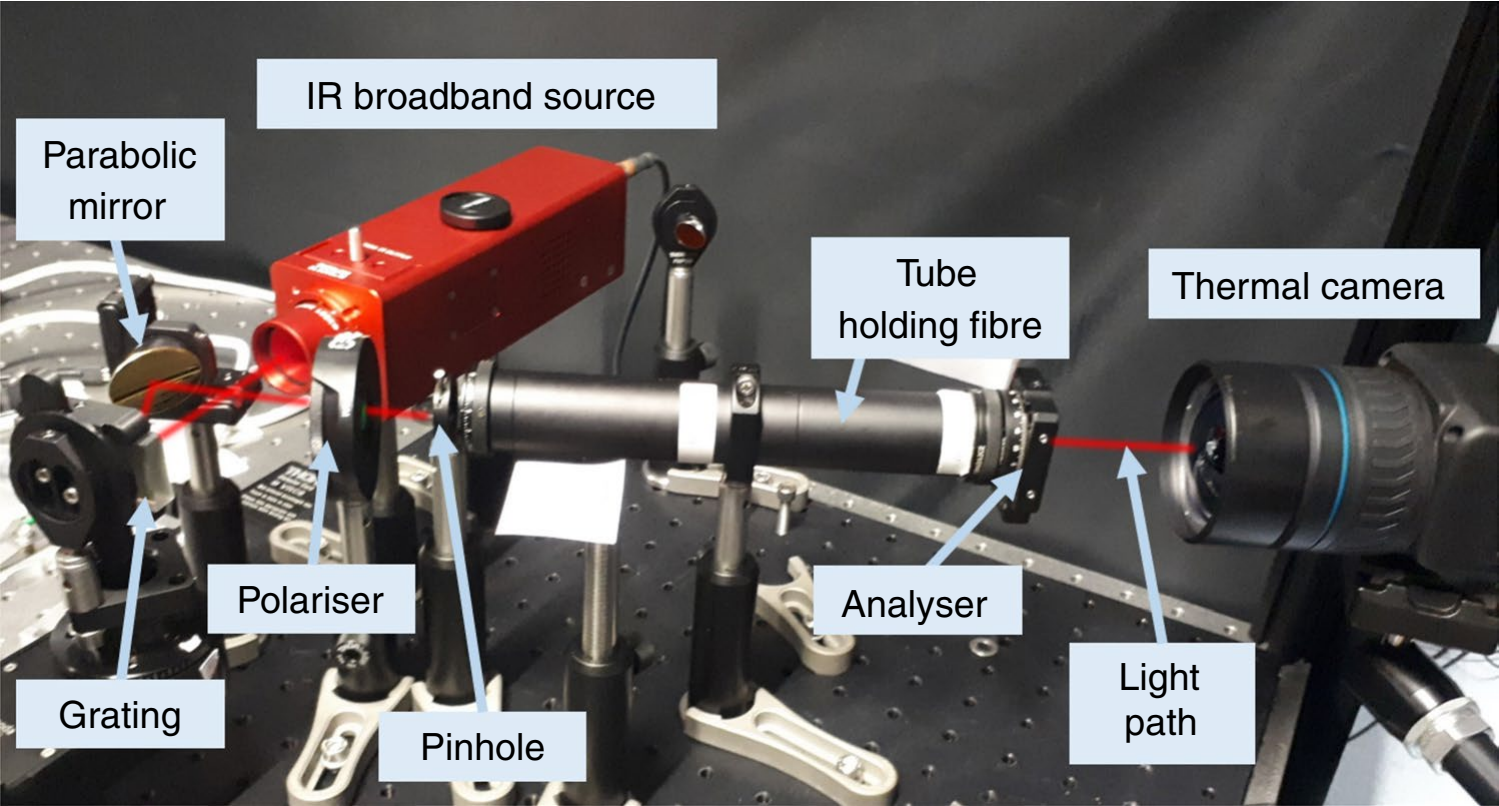
Experimental set-up consisting of IR broadband source, grating, concave mirror, polariser and analyser, tube holding fibre and thermal camera

We built a simple experimental set-up to show the polarisation effect on IRT measurements through the bundle fibre. The infrared light source was a compact stabilised and collimated broadband silicon carbide globar thermal source (Thorlabs SLS203L). The light emitted was of a constant intensity with a blackbody radiation spectrum of 500–9000 nm and with peak spectral power at approximately 2400 nm. The broadband collimated beam emitted from the source was first incident on a grating, from which the zero-order reflection is directed and focused on the bundle fibre entrance via a parabolic mirror as shown in Fig. 1. The cross-polarisation configuration is built around the bundle fibre with a linear broadband polariser at the fibre entrance‚ so-called polariser‚ and a second one‚ so-called analyser‚ placed at the bundle fibre exit. The fibre ends were held in-line with the beam path, and a rigid tube was placed around the fibre to minimise any bending or curvature in the fibre. This was surrounded by an additional opaque tube with ends closed around the fibre, to prevent the interaction of any external light sources with the fibre. The orientations of the two polarisers will be described in further detail below. Upon leaving the analyser, the beam is then incident on a thermal camera. The camera used in this work is a FLIR A655sc with uncooled microbolometer detector. A thermal image of the fibre end is recorded in addition to the numeric temperature values of each pixel in the image.

The cross-polarisation experiment was carried out with two polariser orientations. In the first configuration, the polariser was set to 180° (horizontal), and the analyser was rotated from 80° through 190° in steps of 2°. This rotation of the analyser ensured that the transmission axes of the polariser–analyser pair passed through both a parallel (polariser: 180°; analyser: 180°) and a perpendicular that is so-called “cross-polarised” (polariser: 180°; analyser: 90°) alignment. An additional 10° was recorded on either side of the parallel and perpendicular alignments. In the second configuration, the polariser was set to 270° (vertical), and the analyser was rotated from 170° through 280° in steps of 2°. Again, this ensured both a parallel and cross-polarised alignment of the transmission axes of the polariser–analyser pair.

This experiment was also carried out for each of the two configurations without the fibre in place.

### Analysis

**Fig. 2:**
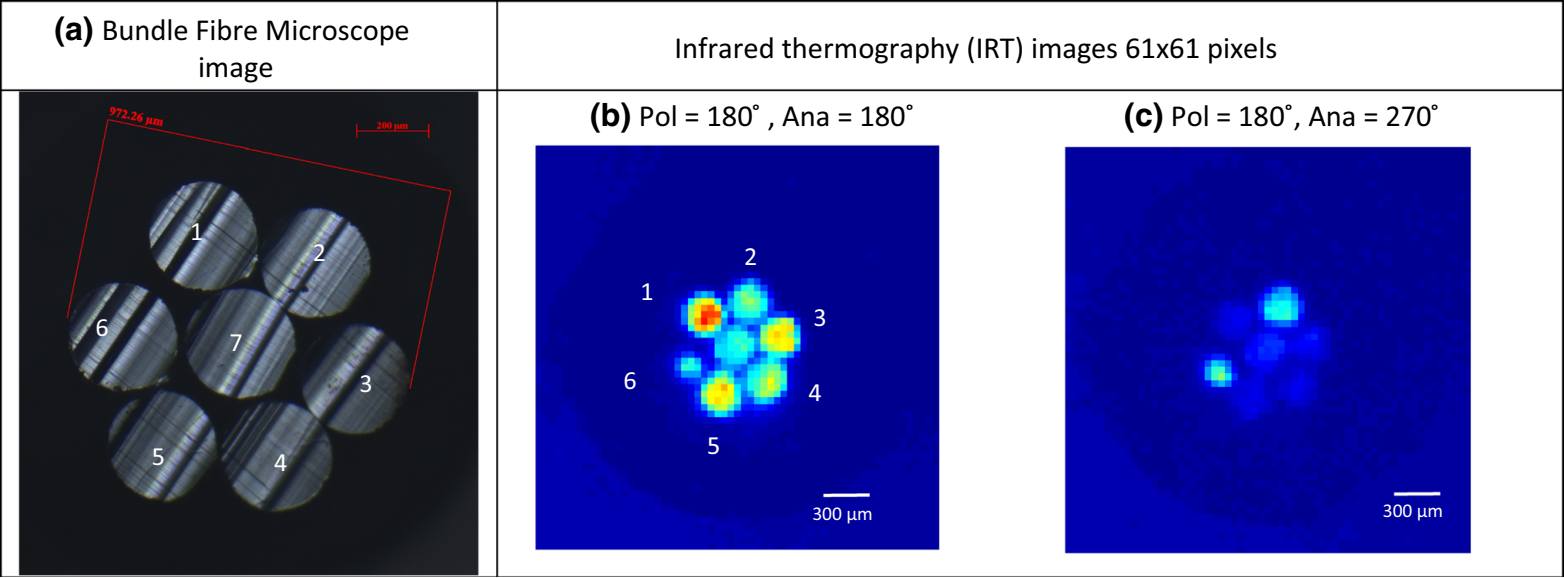
Bundle fibre images: **a** Microscope image showing geometrical bundle fibre configuration, **b** and **c** showing horizontal configuration (Polariser = 180°) IRT images for two different analyser positions: parallel polarisers PP (Analyser = 180°) and cross-polarisers CP (Analyser = 270°), respectively

After collection, the dataset of the pixelated temperature values of each image was imported into MATLAB for analysis. As the positions of the fibres in each image were constant, the set of images for each configuration were examined to determine the pixel locations of each fibre. For the analysis, fibre ends were taken to be perfectly circular and of equal size. By identifying the pixels corresponding to each fibre in the bundle, it was possible to generate a script to extract the required quantitative information from each image for analysis. The maximum recorded value of each fibre exit area was thus assigned to each of the fibres in the bundle as the transmitted fibre exit temperature (TFET). The average temperature across the fibre exit area can be an alternative criterion for analysis. It displays the same behaviour, but with lower temperature values overall. Figure 2 shows (a) bundle fibre geometrical configuration image taken by Zeiss optical microscope, (b) and (c) are the IRT images using FLIR close-up camera lens, in horizontal polariser configuration (Pol = 180°) for two different analyser positions: parallel polarisers (PP) (Ana = 180°) and cross-polarisers (CP) (Ana = 270˚), respectively. Note that the maximum measured temperatures on both cases of PP (a) and CP (b) are 50.65 C and 41.13 C, which visibly demonstrate a net temperature change over the IRT images following polarisation configuration.

**Fig. 3:**
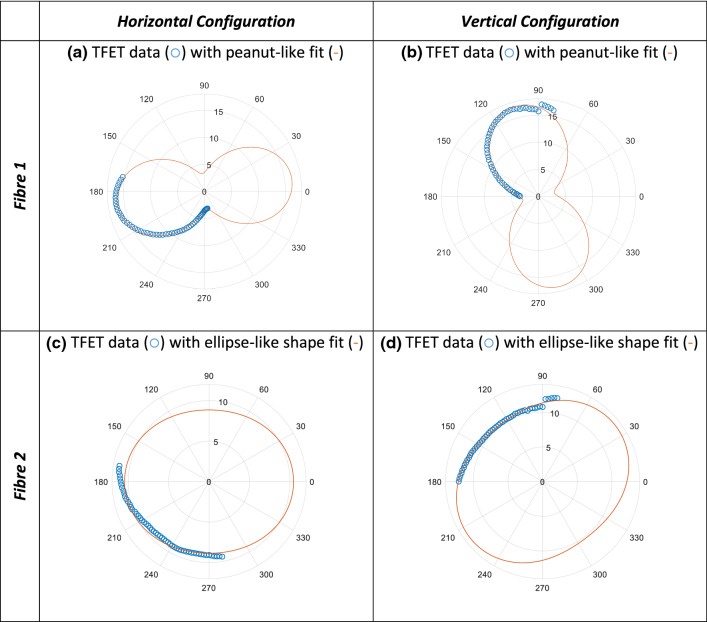
Collected transmitted fibre exit temperature TFET of fibre 1 and fibre 2 for the horizontal configuration as in (**a**) and (**c**), and vertical one as in (**b**) and (**d**), respectively

Figure 3 shows the collected TFET data (blue data point markers: ○) of fibres 1 and 2 only. Their distinguished shape fits are shown in red (-). Two shapes have been identified: the peanut-like as in (a, b) and ellipse-like shapes as in (c, d) for both horizontal and vertical polariser configurations. As shown, the data of rotated analyser are enough to construct the fits of the above-mentioned shapes with good determination of the TFET extrema, but some difficulties arise concerning the different shapes axes-rotation determination. We think a full revolution of collected data measurements with accurate regular rotation increments can help very much in the determination of shape-axis rotation.

## Results and discussion

### Thermal retention in fibres

**Fig. 4:**
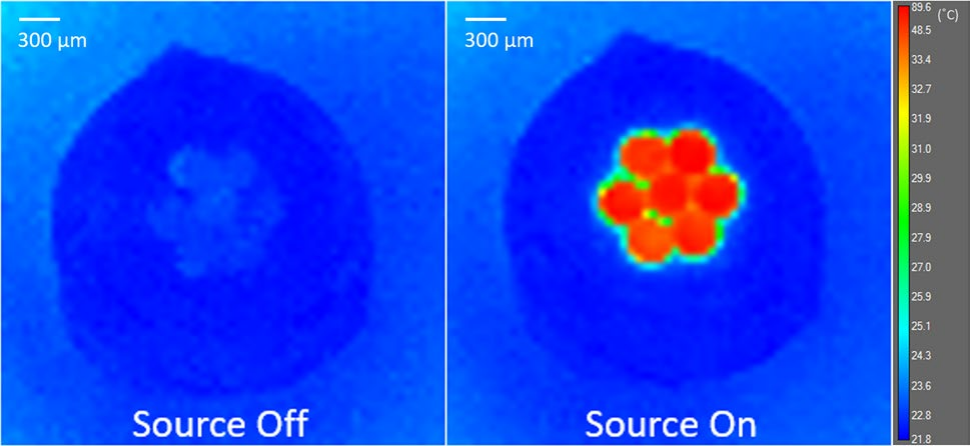
Bundle fibre—source off; Bundle fibre—source on. Colour scale ranges from 21.8 to 48.5 °C. All temperatures > 48.5 °C shown as red

As a preliminary work, the first course of action was to ensure that no heat retention was taking place in the fibres within the time scale of the measurements. It was confirmed that, by repeatedly switching the source on and off, the temperatures detected arose from the transmission of light and not from any residual emission of stored heat within the fibre bundle, see Fig. 4. It is also clear from this assessment that the bundle fibre is capable of transmitting and guiding the infrared light which can be clearly seen by the MIR camera. The same colour scale is used in both images. To enable distinction of low-temperature differences, colour scale ranges from 21.8 to 48.5 °C. All temperatures greater than 48.5 °C are shown as red. Maximum temperature of 89.6 ± 2.0 °C is recorded without polariser and analyser in place.

### Polarisation measurements in fibres

**Fig. 5:**
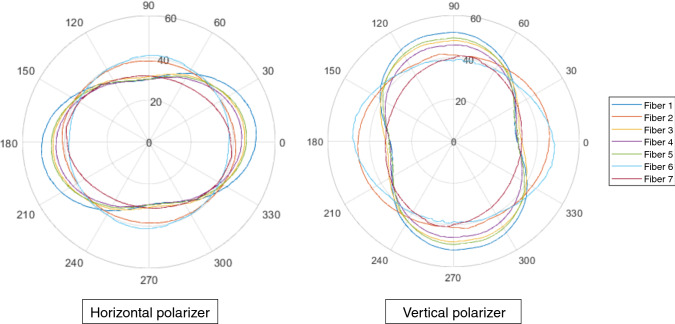
Polar plots of TFET temperatures changing through analyser rotation for each individual fibre in the bundle for horizontal and vertical configuration. Analyser orientation (°) is shown along the angular axes while the radial axes display TFET temperature (°C)

The TFET temperatures for all seven individual fibres of the MIR imaging fibre bundle are shown simultaneously in Fig. 5. The figures show the TFET results for both the horizontal and vertical configurations while rotating the analyser over the corresponding ranges as described in Table 1. The TFET temperatures are displayed along the radial axes, while analyser orientation is displayed along the angular axes. The resulting polar plots showing two different groups of shapes: Group I composed of fibres (1, 3, 4 &5) with peanut-like shape, and Group II composed of the fibres (2, 6 & 7) with elliptical-like forms.

**Table 1:** Summary of polariser, analyser orientations for horizontal and vertical configurations

	Horizontal configuration		Vertical configuration	
	Polariser (Pol)	Analyser (Ana)	Polariser (Pol)	Analyser (Ana)
Start (°)	180	80	270	170
End (°)	180	190	270	280
Rotation steps (°)	0	2	0	2
Parallel alignment (°)	180	180	270	270
Perpendicular alignment (°)	180	90	270	180

Qualitatively, the polar plot shapes as shown in Fig. 5 are dependent on the relative orientation of the polariser–analyser system. For a completely polarised light source, the minimum TFET values should occur with cross-polarisation (perpendicular alignment) of the linear polariser and analyser following Malus’ Law. The converse is also true, with the maximum TFET values arising when the linear polariser and analyser are in parallel alignment. Thus, any deviation of the TFET extrema from this indicates an unpolarising effect resulting from the transmission of the B-MIR light through the fibres of the imaging bundle.

Figure 5 shows that the TFET values for each fibre end differ, with a variation of over 10 °C between the fibre ends of the bundle. This is evident despite a constant intensity of infrared light incident on the fibres in the bundle. However, despite the variation in TFET, the angle at which the TFET maxima occur shows some consistency. The majority of the fibres reach their maximum TFET when the analyser is approximately parallel to the polariser and reach their minimum TFET values when the polariser and analyser are close to cross-polarisation. This behaviour is more pronounced in Group I fibres. The TFET maxima of fibres in Group I appear to occur at angles +6° to +8° beyond parallel. However, as previously noted, the rotation of the symmetry axis of the polarisation plots cannot be accurately determined from this data. The accuracy of each angular position during manual rotation is limited ($\pm$ 1°), with potential additional inaccuracies ($\pm 2^\circ )$ in the initial polariser–analyser angular alignment. Similarly, the axis of rotation of Group II fibres appears to occur at angles of up to $\pm$ 10° to parallel. The accuracy of the angle of rotation in the case of Group II fibres is lowered further, as with polar plot shapes closer to circle form, the orientation information is ambiguous. We believe that taking full 360° rotation measurements (instead of a quarter revolution) can better determine the symmetry axis of the polarisation polar plot shapes, which will consequently increase the accuracy of symmetry axis rotation angle.

Overall and noteworthy to emphasise that the two groups of shapes remain unchanged, i.e., the same fibres have the shapes, when switching the polariser from horizontal to vertical position. This clearly indicates that the polarisation behaviour, i.e., the polar plot shape, is a *pure fibre nature-related polarisation property*. In consequence, this allows us to classify the individual B-MIR fibres into two distinguished categories as follows: Group I represents the fibres with high polarisation sensitivity and Group II the fibres show very slight polarisation dependency.

As the direct and simplest criterion to quantify the effect of the fibre nature on polarisation for IRT applications, we examine the difference between the maximum and minimum TFET values for each fibre as the analyser is rotated from parallel to cross-polarisation.

The polarisation temperature difference is defined for both the horizontal and vertical configurations as${\text{PTD }} = {\text{TFET}}_{ \hbox{max} } - {\text{TFET}}_{ \hbox{min} }$. The PTD analysis results are summarised in Table 2. The PTD uncertainty is determined by the FLIR A655sc instrument accuracy (± 2.0 °C) and is calculated as ${\text{uncertainty}}\left( {\text{PTD}} \right) = \sqrt {{\text{uncertainty}}({\text{TFET}}_{ \hbox{max} } )^{2} + {\text{uncertainty}}({\text{TFET}}_{ \hbox{min} } )^{2} } =$ ± 2.8 °C. The PTD analysis results are summarised in Table 2. The Group I fibres have PTD values in the range 14.6 ± 2.8 °C to 22.1 ± 2.8 °C, which are higher than of those of Group II that range 3.1 ± 2.8 °C to 8.6 ± 2.8 °C. The origin of the polarisation in AgClBr-PolyC B-MIR fibres is as yet unknown and requires further studies.

**Table 2:** Summary of polarisation difference temperature PTD of each individual B-MIR fibre in the imaging bundle

Fibre number	Horizontal configuration PTD/°C	Vertical configuration PTD/°C
Fibre 1	21.9 ± 2.8	22.1 ± 2.8
Fibre 2	3.1 ± 2.8	5.2 ± 2.8
Fibre 3	15.2 ± 2.8	16.3 ± 2.8
Fibre 4	14.6 ± 2.8	15.8 ± 2.8
Fibre 5	16.8 ± 2.8	19.0 ± 2.8
Fibre 6	3.6 ± 2.8	10.2 ± 2.8
Fibre 7	8.6 ± 2.8	8.6 ± 2.8

## Conclusions

In this work, we demonstrate the performances of a simple home-built set-up for studying the polarisation changes in guided infrared thermography IRT using silver halide poly-crystalline AgCl_1−x_Br_x_ (0 ≤ *x*≤1) broadband mid-infrared fibre bundle. The results presented here show clear polarisation dependency of IRT temperature measurements with B-MIR fibres. This polarisation can be identified easily by the analysis of the IRT images recorded at the fibre exit with a close-up lens. This method also enables a simple way to classify the B-MIR fibre following their polarisation sensitivity from highly to slightly sensitive to the thermal source radiations. Future work will include the modelling of light propagation through the B-MIR fibres using finite element analysis. This will provide enhanced understanding of the interaction of the light with the material and structure of the fibre and may lead to new potential applications, in particular, for predictive, preventive and personalised medicine as well as additive manufacturing.
